# Supplementary material for: Modeling neutral viral mutations in the spread of SARS-CoV-2 epidemics
Source: PLoS One. 2021 Jul 29;16(7):e0255438. doi: 10.1371/journal.pone.0255438 (PMC8321105; doi:10.1371/journal.pone.0255438)
Supplement: S1 Appendix — (PDF) [file pone.0255438.s001.pdf]

Modeling neutral viral mutations in the spread of SARS-CoV-2 epidemics

Vitor M. Marquioni[1], Marcus A. M. de Aguiar[1*]

**1** Instituto de Física “Gleb Wataghin”, Universidade Estadual de Campinas - UNICAMP, Campinas, SP, Brazil

* aguiar@ifi.unicamp.br

# 1 Appendix A: Simulation parameters

The network simulations follow the model proposed in reference [1], and the parameters are displayed in Table 1.

| Parameter | Value |
|---|---|
| $R_0$ | 2.4 [2] |
| Average Symptoms Duration $\tau_0$ | 14 days [3, 4] |
| Networks Average Degree $D$ * | 100 [1] |
| Incubation Time Distribution $\mathcal{P}(\tau)$ | $\Gamma(6.25, 25/26)$ [5] |
| Mutation Rate $\mu$ | 0.001 substitution per base, per year [6, 7] |
| Genome Size $B$ | 29900 bases [2] |

**Table 1. Simulation Parameters.** The number of nodes in each simulation is described properly.
*This is the input average degree for the networks construction, but the actual value for each realization fluctuates. For the communities simulations, this is the parameter for constructing each isolated network, as also for the control case $p = 0$.

For the numerical solution of mean-field approaches, following the SEIR model

$$\begin{aligned} \dot{S} &= -\beta SI/N \\ \dot{E} &= \beta SI/N - \sigma E \\ \dot{I} &= \sigma E - \gamma I \\ \dot{R} &= \gamma I \end{aligned} \tag{A1}$$

we have used the following parameters: $R_0 = 2.4$, $\gamma = 1/14$ day$^{-1}$; $\beta = R_0\gamma$ and $\sigma = 1/\langle t_i\rangle$, where $\langle t_i\rangle$ is the mean period of incubation, averaged over the distribution from Table 1 [1].

# 2 Appendix B: Analytical calculations

Our goal is to derive a recurrence equation for the average genetic distance, i.e., given the distance $d_t$ at time $t$, we aim to calculate the distance $d_{t+1}$ at time $t+1$. The idea is to calculate $d_{t+1}$ as a weighted average, where the weights are the number of pairs that are distanced by a certain amount. In a SEIR model, every iteration starts with a given number of recovered ($R_t$), infected ($I_t$) and exposed ($E_t$) individuals. When an individual recovers, its infecting virus stops to spread and to evolve, and we call it a

*final virus*. There are $R_t$ final viruses at the beginning of a given iteration. Viruses infecting Exposed individuals can mutate during this iteration. However, viruses in Infected individuals can either evolve and mutate in this time step or not, since their hosts might recover. The latter become final and are counted as $r_t$. Infected individuals can also spread the virus, which replicate before evolving or becoming final. Such *offspring* ($x_t$) increase the number of viruses in Exposed individuals in the next iteration, when they will be allowed to evolve.

At the beginning of iteration $t+1$, there are $(R_t + E_t + I_t)(R_t + E_t + I_t - 1)/2$ pairs of viruses sharing an average distance equal to $d_t$, but along the iteration some of the distances may increase by a certain amount to be calculated, as also new viruses may arise. Therefore,

$$d_{t+1} = \frac{1}{Z'_t}\left(d_t \frac{(R_t + E_t + I_t)(R_t + E_t + I_t - 1)}{2} + \text{Increases} + \text{Offspring}\right), \quad \text{(A2)}$$

where $Z'_t$ is a normalization factor, which counts the total number of pairs at the end of iteration $t+1$,

$$Z'_t = \frac{(R_t + E_t + I_t + x_t)(R_t + E_t + I_t + x_t - 1)}{2}. \quad \text{(A3)}$$

If the mutation rate is zero and no new infections occur ($x_t = 0$) the "Increases" term and the "Offspring" term are equal to zero, and $d_{t+1} = d_t$, as expected.

In the following two subsections, we shall calculate the "Increases" term and the "Offspring" term, which accounts for the evolution and for the spread, respectively.

## 2.1 Increases

Genetic distances between evolving viruses increase over time. In order to calculate how much these distances increase we first consider that mutations occurring in the same locus of different genomes are unlikely, as well as more than one mutation per locus on a single genome. This approximation holds as long as the epidemic duration $T$ remains sufficiently small, $\mu T \ll 1$. Thus, after one time step, an evolving genome acquires, on average, $B\mu$ mutations. The distance between two evolving genomes will increase, on average, by $2B\mu$ nucleotides after one time step. The distance between viruses in exposed individuals, for example, increases by $2B\mu$ and because there are $E_t(E_t - 1)/2$ pairs of exposed individuals, their evolution along the iteration $t+1$ contributes $2B\mu E_t(E_t - 1)/2$ to the Increases term. On the other hand, the distance between viruses in an exposed and a recovered individual, or an infected individual that recovers, is only $B\mu$, because the latter two do not evolve. There are $E_t(R_t + r_t)$ pairs among these viruses, and thus their contribution to Increases is $E_t(R_t + r_t)B\mu$. We recall that the updates in our model occur in the order "Transmission", "Attempt to Recovery" and lastly, "Genome Evolution". Thus, if an infected individual recovers its virus does not have the chance to mutate.

Therefore, in order to compute the Increases term, we must calculate the average increase in distance between all pairs of viruses and how many pairs of these viruses exist. Table 2 summarizes this information. We obtain

$$\begin{aligned}\text{Increases} =& E_t R_t B\mu + E_t r_t B\mu + (I_t - r_t) r_t B\mu + (I_t - r_t) R_t B\mu + (I_t - r_t) E_t 2B\mu \\ &+ \frac{E_t(E_t - 1)}{2} 2B\mu + \frac{(I_t - r_t)(I_t - r_t - 1)}{2} 2B\mu. \end{aligned} \quad \text{(A4)}$$

**Table 2. Increases in average distance and number of pairs of viruses.**

| Viruses | Number of Pairs | Average Distance Increase |
|---|---|---|
| $(E_t)$ and $(R_t)$ | $E_tR_t$ | $B\mu$ |
| $(E_t)$ and $(r_t)$ | $E_tr_t$ | $B\mu$ |
| $(I_t - r_t)$ and $(r_t)$ | $(I_t - r_t)r_t$ | $B\mu$ |
| $(I_t - r_t)$ and $(R_t)$ | $(I_t - r_t)R_t$ | $B\mu$ |
| $(I_t - r_t)$ and $(E_t)$ | $(I_t - r_t)E_t$ | $2B\mu$ |
| $(E_t)$ and $(E_t)$ | $E_t(E_t - 1)/2$ | $2B\mu$ |
| $(I_t - r_t)$ and $(I_t - r_t)$ | $(I_t - r_t)(I_t - r_t - 1)/2$ | $2B\mu$ |
| $(R_t)$ and $(R_t)$ | $R_t(R_t - 1)/2$ | 0 |
| $(r_t)$ and $(r_t)$ | $r_t(r_t - 1)/2$ | 0 |
| $(r_t)$ and $(R_t)$ | $r_tR_t$ | 0 |

## 2.2 Offspring

The contribution of the new infections to the average distance $d_{t+1}$, the Offspring term, is more tricky. To simplify matters we will assume that an infected individual infects only one susceptible per time step, which is a good assumption if the basic reproduction number $R_0$ is small compared to the average duration of symptoms. Thus, $x_t$ is also the number of individuals who infected a susceptible within the time step $t + 1$, which will be called *ancestors* from now on. Let $D_1$ be the average distance between ancestors and the other viruses at time $t$, and $D_2$, the distance between the exposed and the other viruses. Note that an ancestor may recover and, therefore, not mutate in this time step. The Offspring term is a sum of different contributions between offspring and the other viruses in the population, as explained in detail below.

1. *Genetic distance between offspring and recovered.* The number of pairs is $x_tR_t$. Because offspring do not evolve in the time step they appear, their average distance is $D_1$. Then, its contribution to the Offspring term is $\boldsymbol{x_tR_tD_1}$.

2. *Genetic distance between offspring and exposed.* The number of pairs is $x_tE_t$. Because the exposed evolve, these pairs contribute with $\boldsymbol{x_tE_t(D_2 + B\mu)}$ to the Offspring term.

3. *Genetic Distance between offspring of an infected (ancestor) that does not recover (there are $(I_t - r_t)$ of these individuals) and infected*:

   (a) The distance between an offspring and its ancestor is $B\mu$, since the ancestor evolves. There are $x_t(I_t - r_t)/I_t$ new infections of this type, contributing with $\boldsymbol{x_t((I_t - r_t)/I_t)B\mu}$ to the distance.

   (b) For each offspring there are $I_t - r_t - 1$ infected individuals that did not recover and are not its ancestral. The distance between the offspring and these individuals is $(D_1 + B\mu)$, adding $\boldsymbol{x_t((I_t - r_t)/I_t)(I_t - r_t - 1)(D_1 + B\mu)}$ to the Offspring term.

   (c) The distance between the offspring and individuals that recover is $D_1$, because neither of these viruses evolve in this time step. There are $x_t((I_t - r_t)/I_t)r_t$ pairs of these viruses, adding $\boldsymbol{x_t((I_t - r_t)/I_t)r_tD_1}$ to the Offspring term.

4. *Genetic distance between offspring of infected (ancestor) that recover in this iteration (there are $r_t$ of these individuals) and infected*:

(a) The distance between offspring and its ancestor is zero, because none of them evolve.

(b) The distance between the offspring and the other viruses of type is $D_1$. There are $x_t r_t/I_t$ new infections of this type, contributing $\boldsymbol{(x_t r_t/I_t)(r_t - 1)D_1}$ to the Offspring term.

(c) The distance between offspring and the other infected individuals is $\boldsymbol{(x_t r_t/I_t)(I_t - r_t)(D_1 + B\mu)}$, since the other infected viruses evolve..

5. *Genetic distance between offspring.* Because each ancestor gives rise to only one new infection, this distance equals $D_1$, and once there are $x_t(x_t - 1)/2$ pairs of offspring, this contribution is $\boldsymbol{(x_t(x_t - 1)/2)D_1}$.

6. By summing everything up, we get

$$\begin{aligned}\text{Offspring} = {} & x_t R_t D_1 + x_t E_t (D_2 + B\mu) \\ & + x\frac{(I_t - r_t)}{I_t} B\mu + x_t \frac{(I_t - r_t)}{I_t}(I_t - r_t - 1)(D_1 + B\mu) + x_t \frac{(I_t - r_t)}{I_t} r_t D_1 \\ & + x_t \frac{r_t}{I_t} 0 + x_t \frac{r_t}{I_t}(r_t - 1)D_1 + x_t \frac{r_t}{I_t}(I_t - r_t)(D_1 + B\mu) \\ & + \frac{x_t(x_t - 1)}{2} D_1. \end{aligned} \tag{A5}$$

Putting all these terms together and defining $Z_t \equiv 2Z'_t$ we obtain

$$\begin{aligned} d_{t+1} = {} & \frac{1}{Z_t}\left(d_t(R_t + E_t + I_t)(R_t + E_t + I_t - 1)\right. \\ & + x_t D_1 (x_t - 3 + 2R_t + 2I_t + 2E_t D_2/D_1) \\ & \left. + 2B\mu(E_t + I_t - r_t)(E_t + I_t + R_t + x_t - 1)\right). \end{aligned} \tag{A6}$$

The reason for assigning the distance $D_1$ between infected and other viruses, instead of $d_t$, is that infected individuals represent only a fraction of the viruses in the population, and the distance between them and other viruses grows over time, therefore being above the average $d_t$. The same holds for the exposed individuals.

Although we were not able to analytically find an expression for $D_1$ and $D_2$, we can approximate them as follows. First we assume that $D_2 \approx D_1$. When the epidemic begins, all viruses are infected, so that $D_1 = d_t$. However, the ratio between infected and recovered individuals decreases to zero along the epidemic, making $D_1$ larger than $d_t$. Thus, to first order, it is possible to approximate $D_1 \approx d_t(1 + \epsilon)$, with $\epsilon$ a function of the number of recovered individuals, $R_t/(I_t + E_t + R_t)$ and the average number of mutations $B\mu$. Our simulations showed that the linear function $D_1 = d_t(1 + 2B\mu R_t/(I_t + E_t + R_t))$ works well (considering the parameters in Appendix A), leading to the theoretical result expressed by Eq.(2) from the main text.

## 2.3 Continuum Limit

To achieve the continuum limit we start by substituting $r_t = R_{t+1} - R_t$ and $x_t = E_{t+1} - E_t + I_{t+1} - I_t + R_{t+1} - R_t$ in Eq.(2) from the main text and subtracting $d_t$ from both sides of this equation:

$$\begin{aligned} d_{t+1} - d_t = {} & \frac{1}{Z_t}\left\{2d_t\left(E_{t+1} - E_t + I_{t+1} - I_t + R_{t+1} - R_t\right) \times\right. \\ & \times \left[-1 + B\mu \frac{R_t}{I_t + E_t + R_t}\left(R_{t+1} + R_t + I_{t+1} + I_t + E_{t+1} + E_t - 3\right)\right] \\ & \left. + 2B\mu\left(E_t + I_t + R_t - R_{t+1}\right)\left(E_{t+1} + I_{t+1} + R_{t+1} - 1\right)\right\} \end{aligned} \tag{A7}$$

with

$$Z_t = (E_{t+1} + I_{t+1} + R_{t+1})(E_{t+1} + I_{t+1} + R_{t+1} - 1). \quad \text{(A8)}$$

Then, we consider the first order approximations

$$f_t \approx f(t)$$
$$f_{t+1} \approx f(t) + \dot{f}(t)\Delta t,$$

and once $B\mu$ in the last line of Eq.(A7) is the number of mutations per time step, we replace it by $B\mu\Delta t$

$$\begin{aligned}
&\dot{d}(t)\Delta t = \quad \text{(A9)}\\
&\frac{1}{Z_t}\Big\{2d(t)\Delta t\left(\dot{E}(t) + \dot{I}(t) + \dot{R}(t)\right)\times\\
&\times\left[-1 + B\mu\frac{R(t)}{I(t)+E(t)+R(t)}\left(2R(t) + 2I(t) + 2E(t) + \Delta t(\dot{E}(t) + \dot{I}(t) + \dot{R}(t)) - 3\right)\right]\\
&+ 2B\Delta t\mu\left(E(t) + I(t) - \dot{R}(t)\Delta(t)\right)\left(R(t) + I(t) + E(t) + \Delta t(\dot{E}(t) + \dot{I}(t) + \dot{R}(t)) - 1\right)\Big\} \quad \text{(A10)}
\end{aligned}$$

with

$$\begin{aligned}
Z_t = &(R(t) + I(t) + E(t) + \Delta t(\dot{E}(t) + \dot{I}(t) + \dot{R}(t)))\times\\
&\times(R(t) + I(t) + E(t) + \Delta t(\dot{E}(t) + \dot{I}(t) + \dot{R}(t)) - 1). \quad \text{(A11)}
\end{aligned}$$

Finally, by taking the limit $\Delta t \to 0$ we obtain the continuous time equation.

## 2.4 Multiple Infections

The average distance $d^{(i)}_{root,t}$ between viruses from a lineage and its root is calculated using the same technique discussed above, however it is much simpler, once we only need to calculate the average distance from a kind of virus and the root (a single virus which does not evolve). Using the same notation, but now with a super-index to denote the lineage, we obtain

$$d^{(i)}_{root,t+1} = \frac{1}{Z_t}\left[\left(R^{(i)}_t + E^{(i)}_t + I^{(i)}_t\right)d^{(i)}_{root,t} + E^{(i)}_t B\mu + \left(I^{(i)}_t - r^{(i)}_t\right)B\mu + x^{(i)}_t D^{(i)}_{1,root}\right] \quad \text{(A12)}$$

with $Z_t = (E^{(i)}_t + I^{(i)}_t + R^{(i)}_t + x^{(i)}_t)$ and $D^{(i)}_{1,root}$ being the average distance between infected and the root, which is given (similarly to $D_1$) by

$$D^{(i)}_{1,root} = d^{(i)}_{root,t}\left(1 + 4B\mu\frac{R^{(i)}_t}{E^{(i)}_t + I^{(i)}_t + R^{(i)}_t}\right).$$

The factor 4 is a fit from numerical investigations. The continuum limit is obtained by subtracting $d^{(i)}_{root,t}$ from both sides of Eq.(6) from the main text, applying the continuous approximation for each epidemic curve and taking the limit $\Delta t \to 0$.

# 3 Appendix C: Real genetic evolution algorithm

In order to estimate the real (from genetic data) genetic evolution, we used 55 complete genome sequences collected in China [8]. First, these sequences were ordered and

numbered by its collection date and a matrix of genetic distances $d_{ij}$ between genomes $i$ and $j$ has been constructed. Each pair of sequences were alligned with the Needleman-Wunsch algorithm, with score $+1$ for match and $-1$ for mismatch [9]. Then, the distance between two genomes was computed counting the number of substitutions between the sequences, neglecting *indels*.

We defined a time window $\tau_W = 14 = \tau_0$ days. Thus, every genome collected within $\tau_W$ are considered infected, and the genomes collected before this time window are considered recovered. Now, we calculate the average distance among the infected $d_{I,t}$, recovered $d_{R,t}$ and among infected and recovered $d_{IR,t}$ at the time $t$. Fig.1 shows an example of a distance matrix with a specific time window. Finally, the average distance at time $t$ can be computed as

$$d_t = \frac{d_{I,t}I_t(I_t - 1) + 2d_{IR,t}I_tR_t + d_{R,t}R_t(R_t - 1)}{(R_t + I_t)(R_t + I_t - 1)} \tag{A13}$$

where $I_t$ and $R_t$ are respectively given by $I(t)$ and $R(t)$ described evaluated in the supplemental material.

With this algorithm, we obtained 20 non-overlapping sets of infected genomes. One of these sets contained only one sequence and was not usable; a second set was too far from all other data and was also discarded. Thus, we were able to calculate 18 points (that appear in Fig.4 from the main text) with error bars given by the standard deviation of each set of distances (between infected, recovered and between infected and recovered) at each time $t$.

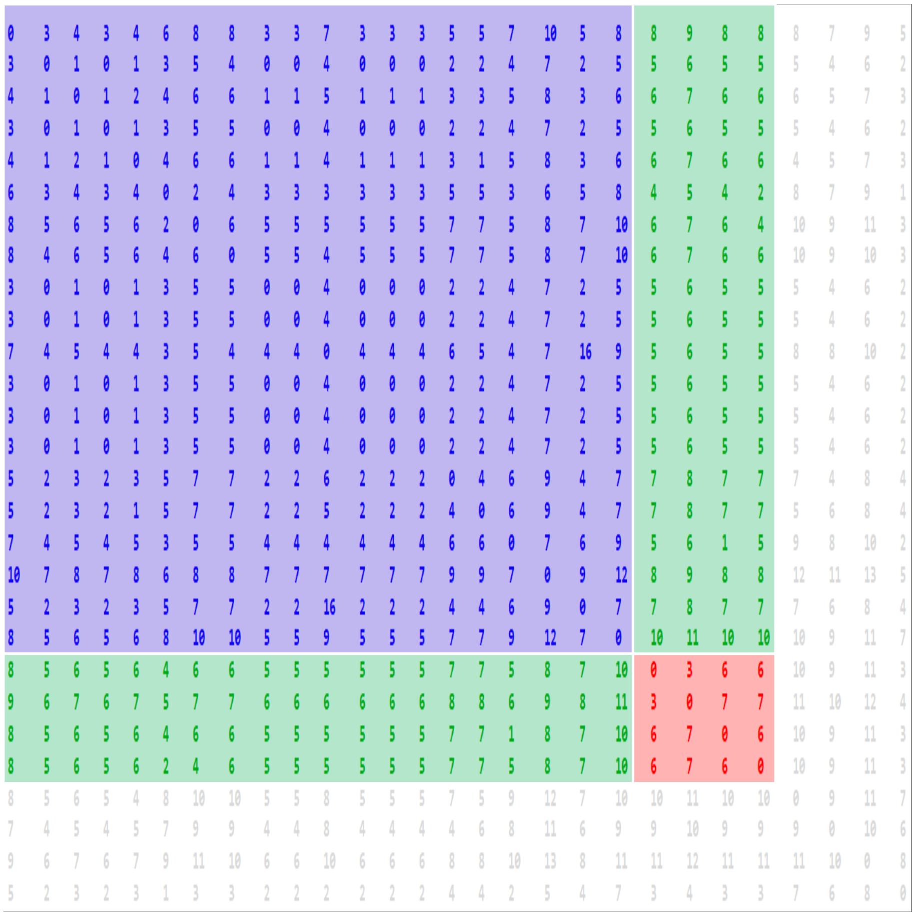

**Fig 1. Example of distance matrix to illustrate the algorithm to infer the genetic evolution.** Every genome collected within a time window $\tau_W$ is considered to belong to an infected individual. The red block shows distances between these viruses. The blue block shows viruses that appeared before the present time window, whose individuals are considered to have recovered. Green blocks are distances between infected and recovered individuals. The remaining entries are distances from viruses that have not appeared yet at that considered time, i.e., they appeared after the considered time window.

# 4 Appendix D: The COVID-19 data from China

We got the Chinese epidemic data from the dataset "Epidemic Data for Novel Coronavirus COVID-19" from Wolfram data repository [10]. Unfortunately, this dataset starts on 22 January (going up to 18 August by the date of our analysis), lacking the previous data. Another concern is about the change in the notification protocols adopted by the Chinese government. On 13 February, the Hubei province started to report not only the positive laboratory tests, but also the clinically diagnosed cases as infected too, appearing a sudden increase in infected curve [11]. We also need to correct the data by including undetected cases.

Firstly, in order to correct the notification problem, we smoothly distribute the sudden increase number of cases among the previous dates. Following reference [11], the

corrected accumulated number of cases $I_{a,c}(t)$ is given by

$$I_{a,c}(t) = I_a(t) + 15133 \frac{\sum_{i=22\text{ Jan}}^{t} I_a(t)}{\sum_{i=22\text{ Jan}}^{13\text{ Feb}} I_a(t)} \tag{A14}$$

for $t \in \{22\text{ Jan}, \ldots, 12\text{ Feb}\}$, where $I_c(t)$ is the accumulated number of cases at date $t$, and $15133 = I_a(13\text{ Feb}) - I_a(12\text{ Feb})$ is the sudden increase due to the changes in the notification protocol.

Now, the undetected cases in China were estimated in reference [12], and also following reference [11], we get

$$I_{a,c'}(t) = \frac{I_{a,c}(t)}{1 - \theta(t)} \tag{A15}$$

for the estimated total number of cases at time $t$, where $\theta$ is the undetected fraction,

$$\theta(t) = \begin{cases} 0.86, \text{ for } t \leq 24\text{ Jan} \\ \textit{linear decrease}, \text{ for } 24\text{ Jan} \leq t \leq 08\text{ Feb} \\ 0.31, \text{ for } t \geq 08\text{ Feb} \end{cases} \tag{A16}$$

This correction is also applied to the recovered curve. However, the Wolfram data distincts recovered $Rec(t)$ from deaths $Dea(t)$, while our theory does not differentiates these numbers. Thus, the number of recovered individuals we must consider is

$$R(t) = \frac{Rec(t) + Dead(t)}{1 - \theta(t)} \tag{A17}$$

and the infected curve is now given as

$$I(t) = I_{a,c'}(t) - R(t) \tag{A18}$$

Fig.2 shows the curves after these corrections. Once we do no have directly access to exposed data, we did not consider exposed individuals, meaning, at this point, that we are dealing with a SIR model without any prejudice to the present theory. However, bad data is an important source of error.

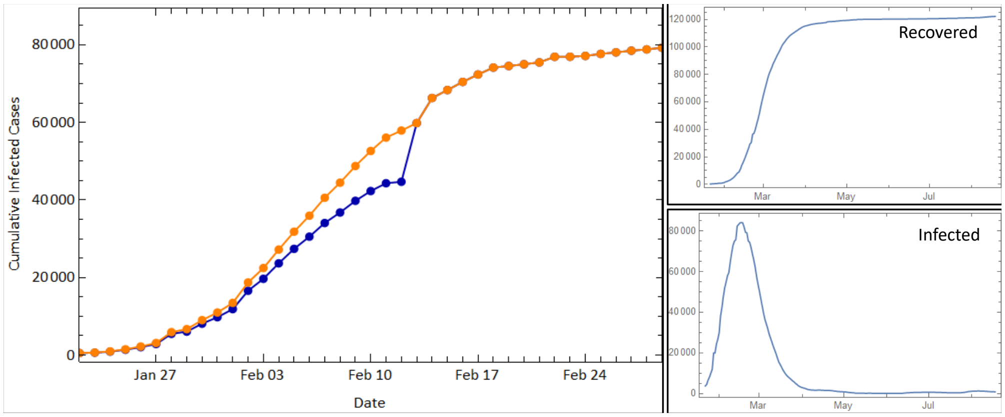


**Fig 2. Chinese epidemic curves after corrections.** The left chart shows the cumulative number of infections in China. The blue curve is the reported number of cases before the smoothness procedure of Eq.(14) and the orange curve is the result of this procedure. The right charts are the recovered and infected curves $R(t)$ and $I(t)$.

Finally, we fit an exponential curve to a few initial data points of $I(t)$ and $R(t)$ and extrapolate it to previous dates. For the $I$-curve, we have adjusted the exponential $e^{a(t-t_0)}$, with fit parameters $a$ and $t_0$, on the first $n_I = 10$ data points and extrapolated it up to the first case $t_0$ days before. With this approach, we found $t_0 = 11$ Dec, which is close to the first case reported by WHO, 08 Dec [13]. For the $R(t)$-curve, we have used the first $n_R = 13$ data points. The numbers $n_I$ and $n_R$ were chosen in order to make the exponential extrapolation makes sense according to WHO estimates of the first case, as also to make $R(t) < I(t)$ in a plausible way.

Now, the curves $R(t)$ and $I(t)$ can be implemented in the recurrence equation and the distance evolution can be estimated, with the first distance $d_0$ equalling zero.

## References


1. Marquioni VM, de Aguiar MAM. Quantifying the effects of quarantine using an IBM SEIR model on scalefree networks. Chaos, Solitons & Fractals. 2020;138:109999. doi:https://doi.org/10.1016/j.chaos.2020.109999.

2. Guo YR, Cao QD, Hong ZS, Tan YY, Chen SD, Jin HJ, et al. The origin, transmission and clinical therapies on coronavirus disease 2019 (COVID-19) outbreak–an update on the status. Military Medical Research. 2020;7(1):1–10.

3. Akhmetzhanov AR, Mizumoto K, Jung Sm, Linton NM, Omori R, Nishiura H. Epidemiological characteristics of novel coronavirus infection: A statistical analysis of publicly available case data. medRxiv. 2020;.

4. Morris DH, Rossine FW, Plotkin JB, Levin SA. Optimal, near-optimal, and robust epidemic control. arXiv preprint arXiv:200402209. 2020;.

5. Backer JA, Klinkenberg D, Wallinga J. Incubation period of 2019 novel coronavirus (2019-nCoV) infections among travellers from Wuhan, China, 20–28 January 2020. Eurosurveillance. 2020;25(5):2000062.

6. Shen Z, Xiao Y, Kang L, Ma W, Shi L, Zhang L, et al. Genomic Diversity of Severe Acute Respiratory Syndrome–Coronavirus 2 in Patients With Coronavirus Disease 2019. Clinical Infectious Diseases. 2020;71(15):713–720. doi:10.1093/cid/ciaa203.

7. Zhao Z, Li H, Wu X, Zhong Y, Zhang K, Zhang YP, et al. Moderate mutation rate in the SARS coronavirus genome and its implications. BMC evolutionary biology. 2004;4(1):21.

8. Wolfram Research. Genetic Sequences for the SARS-CoV-2 Coronavirus; 2020. Wolfram Data Repository `https://doi.org/10.24097/wolfram.03304.data`.

9. Sung WK. Algorithms in bioinformatics: A practical introduction. CRC Press; 2009.

10. Wolfram Research. Epidemic Data for Novel Coronavirus COVID-19; 2020. Wolfram Data Repository `https://doi.org/10.24097/wolfram.04123.data`.

11. Ivorra B, Ferrández MR, Vela-Pérez M, Ramos A. Mathematical modeling of the spread of the coronavirus disease 2019 (COVID-19) taking into account the undetected infections. The case of China. Communications in nonlinear science and numerical simulation. 2020;88:105303.

12. Li R, Pei S, Chen B, Song Y, Zhang T, Yang W, et al. Substantial undocumented infection facilitates the rapid dissemination of novel coronavirus (SARS-CoV-2). Science. 2020;368(6490):489–493.

13. World Health Organization. Coronavirus disease 2019 (COVID-19) Situation Report 37; 2020. https://www.who.int/docs/default-source/coronaviruse/situation-reports/20200226-sitrep-37-covid-19.pdf?sfvrsn=2146841e_2.
