# Supplementary material for: Modeling neutral viral mutations in the spread of SARS-CoV-2 epidemics
Source: PLoS One. 2021 Jul 29;16(7):e0255438. doi: 10.1371/journal.pone.0255438 (PMC8321105; doi:10.1371/journal.pone.0255438)
Supplement: S1 Table — All genomes registered in Wolfram Repository “Genetic Sequences for the SARS-CoV-2 Coronavirus” with complete NucleotideStatus and human Host from China (data accessed 19/08/2020). (PDF) [file pone.0255438.s002.pdf]

S1 Table. All Chinese genome sequences. All genomes registered in Wolfram Repository "Genetic Sequences for the SARS-CoV-2 Coronavirus" with complete *NucleotideStatus* and human *Host* from China (data accessed 19/08/2020) [1].

Accession Number	Collection Date	Length	Geographic Location	Included?	Justification
MN908947	26 Dec 2019*	29903	Wuhan, Hubei**	Y	
MN938384	10 Jan 2020	29838	Shenzen, Guangdong	Y	
MN975262	11 Jan 2020	29891	Wuhan, Hubei**	Y	
MN988668	02 Jan 2020	29881	Wuhan, Hubei**	Y	
MN988669	02 Jan 2020	29881	Wuhan, Hubei**	Y	
MN996527	30 Dec 2019	29825	Wuhan, Hubei	Y	
MN996528	30 Dec 2019	29891	Wuhan, Hubei	Y	
MN996529	30 Dec 2019	29852	Wuhan, Hubei	Y	
MN996530	30 Dec 2019	29854	Wuhan, Hubei	Y	
MN996531	30 Dec 2019	29857	Wuhan, Hubei	Y	
MT019529	23 Dec 2019	29899	Wuhan, Hubei	Y	
MT019530	30 Dec 2019	29889	Wuhan, Hubei	N	MT19530 to MT19532: Might be biased data (sequences from the same researchers, collected at the same day and with quite the same length, with no other informations up to the date we have made the analysis).*
MT019531	30 Dec 2019	29899	Wuhan, Hubei	N	
MT019532	30 Dec 2019	29890	Wuhan, Hubei	N	
MT019533	01 Jan 2020	29883	Wuhan, Hubei	Y	
MT034054	03 Jan 2020	29885	Beijing	Y	
MT039873	20 Jan 2020	29833	Hangzhou, Zhejiang	Y	
MT039874	22 Jan 2020	29858	Hangzhou, Zhejiang**	Y	
MT049951	17 Jan 2020	29903	Kunming,† Yunnan	Y	
MT079843	22 Jan 2020	29915	Wuhan, Hubei**	Y	MT079843 to MT079854: Might be biased data (probable nosocomial transmission).** Then we have included only one genome.
MT079844	22 Jan 2020	29910	Wuhan, Hubei**	N	
MT079845	22 Jan 2020	29955	Wuhan, Hubei**	N	
MT079846	22 Jan 2020	29903	Wuhan, Hubei**	N	
MT079847	22 Jan 2020	29872	Wuhan, Hubei**	N	
MT079848	22 Jan 2020	29880	Wuhan, Hubei**	N	
MT079849	22 Jan 2020	29904	Wuhan, Hubei**	N	
MT079850	22 Jan 2020	29885	Wuhan, Hubei**	N	
MT079851	22 Jan 2020	30018	Wuhan, Hubei**	N	
MT079852	22 Jan 2020	29891	Wuhan, Hubei**	N	
MT079853	22 Jan 2020	29766	Wuhan, Hubei**	N	
MT079854	22 Jan 2020	29897	Wuhan, Hubei**	N	
MT093631	08 Jan 2020	29860	Beijing†	N	No detailed geographic information available.
MT121215	02 Feb 2020	29945	Shanghai	Y	

MT123290	05 Feb 2020	29891	Guangzhou, Guangdong	Y	
MT123291	29 Jan 2020	29882	Guangzhou, Guangdong	Y	
MT123292	27 Jan 2020	29923	Guangzhou, Guangdong	Y	
MT123293	29 Jan 2020	29871	Guangzhou, Guangdong	Y	
MT135041	26 Jan 2020	29903	Beijing	N	MT135041 to MT135044: Might be biased data (the lengths are all the same). Then we have included only one genome
MT135042	28 Jan 2020	29903	Beijing	N	
MT135043	28 Jan 2020	29903	Beijing	N	
MT135044	28 Jan 2020	29903	Beijing	Y	
MT226610	20 Jan 2020	29899	Kunming, Yunnan†	N	No detailed geographic information available.
MT253696	23 Jan 2020	29781	Hangzhou, Zhejiang	N	MT253696 to MT253710: Might be biased data (cluster of cases*; also they all have the same length). Then we have included only one genome.
MT253697	23 Jan 2020	29781	Hangzhou, Zhejiang	N	
MT253698	23 Jan 2020	29781	Hangzhou, Zhejiang	N	
MT253699	24 Jan 2020	29781	Hangzhou, Zhejiang	N	
MT253700	25 Jan 2020	29781	Hangzhou, Zhejiang	N	
MT253701	21 Jan 2020	29781	Hangzhou, Zhejiang	N	
MT253702	21 Jan 2020	29781	Hangzhou, Zhejiang	N	
MT253703	25 Jan 2020	29781	Hangzhou, Zhejiang	N	
MT253704	25 Jan 2020	29781	Hangzhou, Zhejiang	N	
MT253705	22 Jan 2020	29781	Hangzhou, Zhejiang	N	
MT253706	22 Jan 2020	29781	Hangzhou, Zhejiang	N	
MT253707	25 Jan 2020	29781	Hangzhou, Zhejiang	N	
MT253708	21 Jan 2020	29781	Hangzhou, Zhejiang	N	
MT253709	21 Jan 2020	29781	Hangzhou, Zhejiang	N	
MT253710	21 Jan 2020	29781	Hangzhou, Zhejiang	Y	
MT259226	10 Jan 2020	29868	Wuhan, Hubei	Y	
MT259227	26 Jan 2020	29863	Wuhan, Hubei	Y	
MT259228	26 Jan 2020	29861	Wuhan, Hubei	Y	
MT259229	26 Jan 2020	29864	Wuhan, Hubei	Y	
MT259230	25 Jan 2020	29866	Wuhan, Hubei	Y	
MT259231	25 Jan 2020	29865	Wuhan, Hubei	Y	
MT281577	10 Mar 2020	29903	Fujyang, Anhui	Y	
MT291826	30 Dec 2019	29807	Wuhan, Hubei	Y	
MT291827	30 Dec 2019	29858	Wuhan, Hubei	Y	
MT291828	30 Dec 2019	29858	Wuhan, Hubei	Y	
MT291829	30 Dec 2019	29774	Wuhan, Hubei	Y	
MT291830	30 Dec 2019	29807	Wuhan, Hubei	Y	
MT291831	24 Jan 2020	29872	Beijing	Y	
MT291832	25 Jan 2020	29828	Beijing	Y	

MT291833	28 Jan 2020	29821	Beijing	Y	
MT291834	28 Jan 2020	29865	Beijing	Y	
MT291835	27 Jan 2020	29834	Beijing	Y	
MT291836	29 Jan 2020	29860	Beijing	Y	
MT407649	22 Jan 2020	29833	Hangzhou,† Zhejiang	Y	
MT407650	22 Jan 2020	29821	Hangzhou,† Zhejiang	Y	
MT407651	22 Jan 2020	29822	Hangzhou,† Zhejiang	Y	
MT407652	26 Jan 2020	29835	Hangzhou,† Zhejiang	Y	
MT407653	26 Jan 2020	29835	Hangzhou,† Zhejiang	Y	
MT407654	24 Mar 2020	29817	Hangzhou,† Zhejiang	Y	
MT407655	24 Mar 2020	29817	Hangzhou,† Zhejiang	Y	
MT407656	24 Mar 2020	29835	Hangzhou,† Zhejiang	Y	
MT407657	24 Mar 2020	29776	Hangzhou,† Zhejiang	Y	
MT407658	24 Mar 2020	29770	Hangzhou,† Zhejiang	Y	
MT407659	24 Mar 2020	29828	Hangzhou,† Zhejiang	Y	
MT412134	24 Feb 2020	29867	Zhengzhou, Henan†	N	No detailed geographic information available.
MT446312	05 Feb 2020	29879	Guangzhou, Guangdong	Y	
MT510727	15 Feb 2020	29903	Meizhou, Guangdong†	N	MT510727 and MT510728: Might be biased data (data from familial
MT510728	13 Feb 2020	29903	Meizhou, Guangdong†	N	cluster*). There is also no detailed geographic information available.
MT534630	26 Jan 2020	29845	Changzhou,Jiangsu	Y	
MT568634	25 Feb 2020	29861	Guangzhou, Guangdong	N	MT568634 to MT568641: data from a work presenting different approaches
MT568635	25 Feb 2020	29854	Guangzhou, Guangdong	N	for genome sequencing.**
MT568636	27 Feb 2020	29858	Guangzhou, Guangdong	N	Then, this data might have more errors than the others.
MT568637	25 Feb 2020	29860	Guangzhou, Guangdong	N	
MT568638	25 Feb 2020	29854	Guangzhou, Guangdong	N	
MT568639	25 Feb 2020	29861	Guangzhou, Guangdong	N	
MT568640	25 Feb 2020	29858	Guangzhou, Guangdong	N	
MT568641	25 Feb 2020	29868	Guangzhou, Guangdong	N	
MT622319	23 Jan 2020	29889	Shanghai†	N	No detailed geographic information available.
MT627325	28 Feb 2020	29859	Shanghai†	N	No detailed geographic information available.
NC045512	Dec 2019	29903	Wuhan, Hubei**	N	Identical to MN908947.*

[1]Wolfram Research. Genetic Sequences for the SARS-CoV-2 Coronavirus; 2020. Wolfram Data Repository `https://doi.org/10.24097/wolfram.03304.data`

GenBank information.
*Publication Information.
† Laboratory address.
