# Supplementary material for: Modeling neutral viral mutations in the spread of SARS-CoV-2 epidemics
Source: PLoS One. 2021 Jul 29;16(7):e0255438. doi: 10.1371/journal.pone.0255438 (PMC8321105; doi:10.1371/journal.pone.0255438)
Supplement: S2 Table — All informations according to S1 Table. (PDF) [file pone.0255438.s003.pdf]

S2 Table. Included sequences sorted by Collection Date. All informations according to S1 Table.

Number	Accession Number	Collection Date	Length	Geographic Location
#1	MT019529	23 Dec 2019	29899	Wuhan, Hubei
#2	MN908947	26 Dec 2019	29903	Wuhan, Hubei
#3	MT291829	30 Dec 2019	29774	Wuhan, Hubei
#4	MT291826	30 Dec 2019	29807	Wuhan, Hubei
#5	MT291830	30 Dec 2019	29807	Wuhan, Hubei
#6	MN996527	30 Dec 2019	29825	Wuhan, Hubei
#7	MN996529	30 Dec 2019	29852	Wuhan, Hubei
#8	MN996530	30 Dec 2019	29854	Wuhan, Hubei
#9	MN996531	30 Dec 2019	29857	Wuhan, Hubei
#10	MT291827	30 Dec 2019	29858	Wuhan, Hubei
#11	MT291828	30 Dec 2019	29858	Wuhan, Hubei
#12	MN996528	30 Dec 2019	29891	Wuhan, Hubei
#13	MT019533	01 Jan 2020	29883	Wuhan, Hubei
#14	MN988668	02 Jan 2020	29881	Wuhan, Hubei
#15	MN988669	02 Jan 2020	29881	Wuhan, Hubei
#16	MT034054	03 Jan 2020	29885	Beijing
#17	MN938384	10 Jan 2020	29838	Shenzhen, Guangdong
#18	MT259226	10 Jan 2020	29868	Wuhan, Hubei
#19	MN975262	11 Jan 2020	29891	Wuhan, Hubei
#20	MT049951	17 Jan 2020	29903	Yunnan
#21	MT039873	20 Jan 2020	29833	Hangzhou, Zhejiang
#22	MT253710	21 Jan 2020	29781	Hangzhou, Zhejiang
#23	MT407650	22 Jan 2020	29821	Zhejiang
#24	MT407651	22 Jan 2020	29822	Zhejiang
#25	MT407649	22 Jan 2020	29833	Zhejiang
#26	MT039874	22 Jan 2020	29858	Hangzhou, Zhejiang
#27	MT079843	22 Jan 2020	29915	Wuhan, Hubei
#28	MT291831	24 Jan 2020	29872	Beijing
#29	MT291832	25 Jan 2020	29828	Beijing
#30	MT259231	25 Jan 2020	29865	Wuhan, Hubei
#31	MT259230	25 Jan 2020	29866	Wuhan, Hubei
#32	MT407652	26 Jan 2020	29835	Zhejiang
#33	MT407653	26 Jan 2020	29835	Zhejiang
#34	MT534630	26 Jan 2020	29845	Changzhou, Jiangsu
#35	MT259228	26 Jan 2020	29861	Wuhan, Hubei
#36	MT259227	26 Jan 2020	29863	Wuhan, Hubei
#37	MT259229	26 Jan 2020	29864	Wuhan, Hubei
#38	MT291835	27 Jan 2020	29834	Beijing
#39	MT123292	27 Jan 2020	29923	Guangzhou, Guangdong
#40	MT291833	28 Jan 2020	29821	Beijing
#41	MT291834	28 Jan 2020	29865	Beijing
#42	MT135044	28 Jan 2020	29903	Beijing
#43	MT291836	29 Jan 2020	29860	Beijing
#44	MT123293	29 Jan 2020	29871	Guangzhou, Guangdong
#45	MT123291	29 Jan 2020	29882	Guangzhou, Guangdong
#46	MT121215	02 Feb 2020	29945	Shanghai
#47	MT446312	05 Feb 2020	29879	Guangzhou, Guangdong
#48	MT123290	05 Feb 2020	29891	Guangzhou, Guangdong
#49	MT281577	10 Mar 2020	29903	Fuyang, Anhui
#50	MT407658	24 Mar 2020	29770	Zhejiang
#51	MT407657	24 Mar 2020	29776	Zhejiang
#52	MT407654	24 Mar 2020	29817	Zhejiang
#53	MT407655	24 Mar 2020	29817	Zhejiang
#54	MT407659	24 Mar 2020	29828	Zhejiang
#55	MT407656	24 Mar 2020	29835	Zhejiang
