# Supplementary material for: Modeling neutral viral mutations in the spread of SARS-CoV-2 epidemics
Source: PLoS One. 2021 Jul 29;16(7):e0255438. doi: 10.1371/journal.pone.0255438 (PMC8321105; doi:10.1371/journal.pone.0255438)
Supplement: S3 Table — We have used a 14 days time window, i. e., every sequenced genome within an interval of 14 days were considered as infected ones, while the previous were considered to be recovered. (PDF) [file pone.0255438.s004.pdf]

**S3 Table. Genome information used to calculate points in Fig.5.** We have used a 14 days time window, i. e., every sequenced genome within an interval of 14 days were considered as infected ones, while the previous were considered to be recovered.

| Point Number* | Infected Genomes | Recovered Genomes | Date Interval |
|---|---|---|---|
| #1 | #03 → #19 | #1 → #02 | 30 Dec 2019 → 12 Jan 2020 |
| #2 | #13 → #19 | #1 → #12 | 01 Jan 2020 → 14 Jan 2020 |
| #3 | #14 → #19 | #1 → #13 | 02 Jan 2019 → 15 Jan 2020 |
| #4 | #16 → #19 | #1 → #15 | 03 Jan 2019 → 16 Jan 2020 |
| #5 | #17 → #27 | #1 → #16 | 10 Jan 2019 → 23 Jan 2020 |
| #6 | #19 → #28 | #1 → #18 | 11 Jan 2019 → 24 Jan 2020 |
| #7 | #20 → #45 | #1 → #19 | 17 Jan 2019 → 30 Jan 2020 |
| #8 | #21 → #46 | #1 → #20 | 20 Jan 2019 → 02 Feb 2020 |
| #9 | #22 → #46 | #1 → #21 | 21 Jan 2019 → 03 Feb 2020 |
| #10 | #23 → #46 | #1 → #22 | 22 Jan 2019 → 04 Feb 2020 |
| #11 | #28 → #48 | #1 → #27 | 24 Jan 2019 → 06 Feb 2020 |
| #12 | #29 → #48 | #1 → #28 | 25 Jan 2019 → 07 Feb 2020 |
| #13 | #32 → #48 | #1 → #31 | 26 Jan 2019 → 08 Feb 2020 |
| #14 | #38 → #48 | #1 → #37 | 27 Jan 2019 → 09 Feb 2020 |
| #15 | #40 → #48 | #1 → #39 | 28 Jan 2019 → 10 Feb 2020 |
| #16 | #43 → #48 | #1 → #42 | 29 Jan 2019 → 11 Feb 2020 |
| #17 | #46 → #48 | #1 → #45 | 02 Feb 2019 → 15 Feb 2020 |
| #18 | #47 → #48 | #1 → #46 | 05 Feb 2019 → 18 Feb 2020 |
| #19** | #49 → #49 | #1 → #48 | |
| #20† | #50 → #55 | #1 → #49 | 24 Mar 2019 → 06 Apr 2020 |

*In Fig.4 from the main text, points are numbered from left to right.
**Since there is only one genome in this time window, we cannot estimate a distance among the infected population, so genome #49 was not used.
† This point was not included in Fig.4 because it is lacking more than one month of genetic information between points #18 and #19, therefore the distance among the recovered population cannot be well inferred.
